# Computed tomography-guided percutaneous biopsy of abdominal lesions: indications, techniques, results, and complications

**DOI:** 10.1590/0100-3984.2017.0045

**Published:** 2018

**Authors:** Luiz Henrique de Oliveira Schiavon, Chiang Jeng Tyng, Demian Junklaus Travesso, Rafael Dias Rocha, Ana Carolina Santana Andrade Schiavon, Almir Galvão Vieira Bitencourt

**Affiliations:** 1 MD, MSc, Imaging Department, A.C.Camargo Cancer Center, São Paulo, SP, Brazil.; 2 MD, PhD, Imaging Department, A.C.Camargo Cancer Center, São Paulo, SP, Brazil.; 3 MD, Imaging Department, A.C.Camargo Cancer Center, São Paulo, SP, Brazil.; 4 MD, Radiology Department, Casa da Esperança de Santo André, Santo André, SP, Brazil.

**Keywords:** Tomography, X-ray computed, Image-guided biopsy, Abdominal neoplasms/diagnostic imaging

## Abstract

**Objective:**

To evaluate the performance of computed tomography (CT)-guided percutaneous biopsy of abdominal lesions.

**Materials and Methods:**

This retrospective, single-center study evaluated patients submitted to CT-guided percutaneous biopsy of abdominal lesions at a cancer center, between January 2014 and June 2015. The images and patient medical records were reviewed using a standardized data collection form.

**Results:**

We included 225 procedures performed in 212 patients, of whom 143 (63.5%) had a prior diagnosis of cancer. Of the 225 lesions biopsied, 88 (39.1%) had a suspected primary origin and 137 (60.9%) were suspected metastatic lesions. Complications occurred in only 14 (6.2%), the most common being self-limited bleeding, which occurred in 12 (85.7%) of the 14. The occurrence of complications was not found to be significantly associated with the lesion location, age of the patient, presence of comorbidities, use of a supplementary technique, vascularization pattern, or proximity of the lesion to large vessels. The pathology findings were sufficient for making the diagnosis in 202 cases (89.8%), and the diagnosis was consistent with the clinical suspicion in 132 (58.6%).

**Conclusion:**

The procedure demonstrated a high (approximately 90%) rate of providing a sufficient sample for the diagnosis and a low complication rate, the most common complication being self-limiting bleeding.

## INTRODUCTION

Percutaneous procedures guided by imaging methods, either ultrasound or computed tomography (CT), have been widely used because they are effective and safe, with high accuracy in the diagnosis of neoplastic lesions in different organs^(^^1^^-^^6^^)^. The option of performing the procedure under CT guidance varies among centers, depending on the experience of the interventional radiologists and the imaging modalities available. In general, the important factors to be considered are the location of the lesion, its visibility on CT compared with other modalities, and the type of pathological specimen required.

The CT-guided procedure has several advantages over the ultrasound-guided procedure^(^^7^^-^^10^^)^: higher image resolution; the ability to visualize the abdominal organs and viscera; and better characterization of retroperitoneal structures. The CT-guided procedure can be employed to obtain biopsies from virtually any abdominal organ^(^^11^^)^. They are minimally invasive procedures that allow a histological diagnosis to be made, as well as being associated with lower morbidity and mortality than are surgical biopsies. However, there have been few studies evaluating the performance of CT-guided percutaneous biopsy or the impact that clinical and radiological factors related to the procedure have on the success rate and complication rate.

The aim of this study was to evaluate the performance of the procedure in obtaining biopsies of abdominal lesions, through the evaluation of its indications, techniques, results, and complications.

## MATERIALS AND METHODS

This was a retrospective, descriptive, single-center study, involving patients who underwent CT-guided percutaneous biopsy of lesions in the abdominal cavity, at a cancer center, between January 2014 and June 2015, and based on the analysis of medical records and the review of images acquired during the procedure. The study was approved by the local research ethics committee before the collection of data.

We employed a standardized data collection form including the following: clinical and demographic characteristics of the patient; clinical suspicion and indication for the procedure; imaging characteristics of the lesion to be biopsied; the technique used in performing the biopsy; the occurrence of complications related to the procedure; the pathological findings; and follow-up data. Patients who sought treatment at our hospital only for the procedure and did not undergo follow-up evaluations at our center were excluded, as were those for whom the procedure documentation was incomplete.

**Figure 1:**
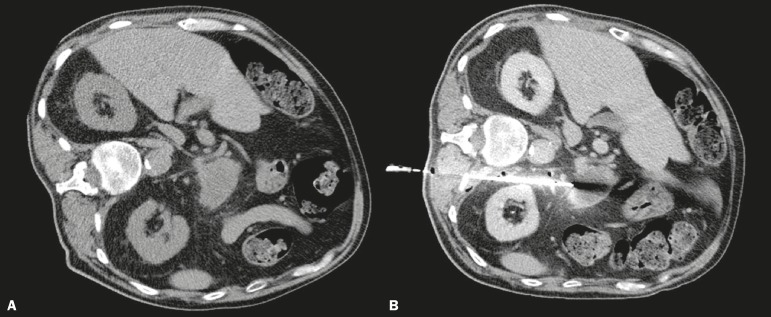
CT-guided percutaneous biopsy using direct access. **A:** Non-contrast-enhanced axial CT scan of the abdomen showing an expansile lesion in the pancreatic body. **B:** Contrast-enhanced axial CT scan of the abdomen showing the path of the coaxial needle through a posterior paravertebral approach.

**Figure 2:**
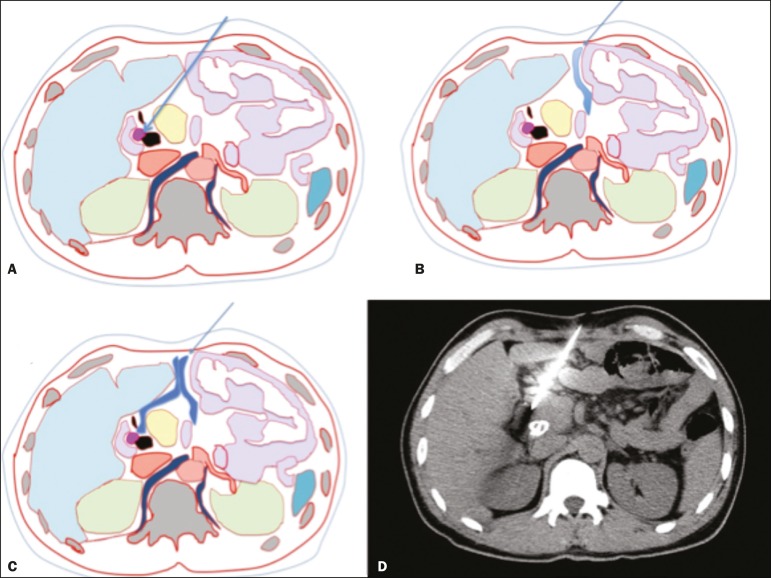
CT-guided percutaneous biopsy using the hydrodissection technique. **A-C:** A schematic CT image of the thorax showing the path from the skin to the lesion in the head of the pancreas, with interposition of intestinal loops along the needle path (**A**), which are displaced after administration of liquid (**B,C**), allowing the needle to be advanced safely. **D:** Axial CT scan showing proper positioning of the needle in the lesion to perform the biopsy. (Images courtesy of Dr. João Paulo Kawaoka Matsushita Jr.).

**Figure 3:**
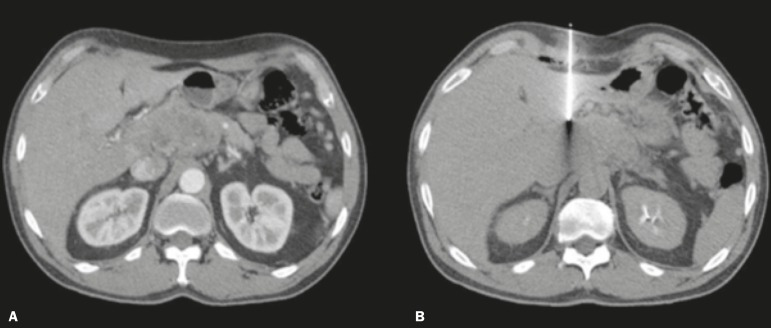
CT-guided percutaneous biopsy using the trans-organ approach. **A:** Contrast-enhanced axial CT scan of the abdomen showing an expansile lesion in the pancreatic head and body. **B:** Axial CT scan of the abdomen, showing the path of the coaxial needle through the liver to the pancreatic lesion.

Procedures were performed in a helical CT scanner (Hispeed; GE Medical Systems, Milwaukee, WI, USA) or in a multidetector CT scanner (Brilliance CT 16; Philips Healthcare, Koninklijke, The Netherlands). The ideal body position to perform the biopsy varies from patient to patient. The preferred position was that which made the patient most comfortable during the procedure. An experienced, board-certified interventional radiologist and a resident in interventional radiology, under supervision, performed the procedures. All biopsies were performed with the coaxial technique. We planned the biopsy approach using pre-procedure images, acquired after intravenous contrast administration when necessary. In cases in which direct access to the lesion (Figure 1) was not possible with the coaxial needle, alternative techniques, such as hydrodissection (Figure 2), pneumodissection, and a trans-organ approach (Figure 3), were used^(^^11^^,^^12^^)^.

Statistical analysis was carried out with the Statistical Package for the Social Sciences, version 16.0 (SPSS Inc., Chicago, IL, USA). Descriptive analysis was performed to calculate absolute and relative frequencies, as well as means and standard deviations, for the study variables. The normality of the distribution of the variables was evaluated by the Shapiro-Wilk test. Dichotomous variables were compared using Student's t-test or nonparametric Mann-Whitney U test. Analysis of variance or the nonparametric Kruskal-Wallis test was used for the comparison of variables with three or more possible values. Categorical variables were examined using Pearson's chi-square test with Yates correction or Fisher's exact test, to evaluate statistical significance. Values of *p* ≤ 0.05 were considered statistically significant.

## RESULTS

### Clinical and demographic characteristics of the patients

A total of 212 patients were evaluated, of whom 116 (51.6%) were male and 109 (48.4%) were female. The mean age of the male patients was 60.7 ± 14.0 years (range, 23-87 years), compared with 55.6 ± 15.0 years (range, 19-104 years) for the female patients. Of the 212 patients, 13 underwent two procedures and 199 underwent one procedure. Therefore, a total of 225 procedures were analyzed.

One hundred and forty-three patients (63.5%) had a history of cancer, the most common type being breast cancer, which accounted for 23 cases (16.0%), followed by pancreatic cancer, in 20 cases (13.9%), and colon cancer, in 18 (12.6%). Fourteen patients (9.8%) had previously been diagnosed with more than one type of cancer.

### Clinical suspicion and imaging characteristics of the lesion to be biopsied

Of the 225 lesions biopsied, 88 (39.1%) were suspected of being primary lesions and 137 (60.9%) were suspected of being metastatic lesions. As shown in Table 1, 105 (46.7%) of the lesions were located in the liver. The mean lesion size was 4.8 ± 3.4 cm (range, 0.7-17.5 cm). Nearly half of the lesions were between 2 cm and 5 cm in size.

**Table 1:** Characteristics of lesions and techniques used for CT-guided percutaneous biopsies (n = 225).

Characteristic	N	%
Location		
Liver	105	46.7
Retroperitoneum	43	19.1
Pancreas	29	12.9
Kidney	19	8.4
Adrenal gland	7	3.1
Spleen	4	1.8
Other	18	8.0
Lesion size (cm)		
< 2	34	15.1
2-5	111	49.3
5-10	60	26.7
> 10	20	8.9
Biopsy technique		
Direct access	110	93.3
Hydrodissection	3	1.3
Pneumodissection	2	0.9
Trans-organ approach	10	4.4

### Biopsy technique

The mean distance between the surface of the skin and the target lesion (needle path) was 8.0 ± 2.9 cm (range, 2.2-15 cm). In 15 biopsies (6.7%), it was not possible to access the lesion with the coaxial needle and an additional technique was therefore required, typically a trans-organ approach (Table 1). In 13 procedures (5.8%), the biopsy was planned on the basis of images acquired with other methods: PET/CT, in 8; and magnetic resonance imaging, in 5.

### Presence of complications related to the procedure

Complications occurred after 14 (6.2%) of the 225 procedures analyzed. The most common complication was bleeding, which was observed in 12 (85.7%) of those 14 procedures. Pneumothorax and pancreatitis occurred after one procedure (7.1%) each. In 10 (71.4%) of those cases, the complications were classified as mild and the patient was discharged on the same day, after supportive care. In four cases (28.6%), the complications were classified as moderate or severe complications, including the following: a subcapsular hepatic hematoma, which required admission to the intensive care unit; a large subcapsular splenic hematoma, which evolved to splenectomy; mild to moderate peripancreatic bleeding, which required hospitalization but had a favorable evolution after a few days; and pancreatitis, which resulted in the only procedure-related death in our sample (corresponding to a procedure-related mortality rate of 0.4%), due to complications of the infectious condition. Most of the complications were observed after biopsies of liver lesions (Table 2). The occurrence of complications was not found to be significantly associated with the location of the lesion, the age of the patient, the presence of comorbidities (for example, diabetes), the use of a supplementary technique, the vascularization pattern, or the proximity of the lesion to large blood vessels.

**Table 2:** Incidence of complications in CT-guided percutaneous biopsies of abdominal lesions (n = 14), by organ.

Organ/space	Number of complications	Percentage of lesions in that location	Percentage of all lesions
Liver	6	5.7	2.7
Kidney	1	5.3	0.4
Pancreas	3	10.3	1.3
Spleen	2	50.0	0.9
Adrenal gland	0	0.0	0.0
Retroperitoneum	1	2.3	0.4
Other	1	5.6	0.4

### Pathological findings and follow-up

The biopsy specimen was sufficient for the diagnosis in 202 (89.8%) of the 225 procedures analyzed. The pathology showed benign lesions in 73 biopsies (32.4%), malignant metastatic lesions in 90 (40.0%), and primary malignant lesions in 62 (27.6%). The diagnosis was concordant with the suspicion in 132 cases (58.6%), and a second biopsy was required in 29 (12.9%). In six cases (2.7%), the patient was referred for surgery because the diagnosis based on the pathological analysis of the percutaneous biopsy sample was inconsistent with the clinical suspicion. However, the percutaneous biopsy findings were confirmed in all six surgical procedures.

In 23 procedures (10.2%), the sample was considered insufficient for diagnosis. In five (21.7%) of those 23 cases, a second percutaneous biopsy was performed and was successful; in seven cases (30.4%), the patients underwent a surgical procedure, resulting in diagnoses of malignancy in six and benignity in one. In 11 cases (47.8%), the patients did not undergo additional invasive diagnostic tests, for several reasons: death, in three patients (attributed to the advanced stage of the disease in two and to the procedure, as previously mentioned, in one); referral for systemic treatment because of high suspicion of the lesion being metastatic and a high number of lesions, in three patients; not being considered a candidate for a second invasive procedure, in two patients; the biopsy being suspended due to stability of the lesions and a low suspicion of malignancy, in two patients; and abandonment of follow-up treatment, in one patient. Table 3 describes the incidence of insufficient sample collection, by organ.

**Table 3:** Incidence of insufficient samples in the first CT-guided percutaneous biopsy of abdominal lesions (n = 23), by organ.

Organ/space	Number of biopsies with insufficient samples	Percentage of biopsies of that location	Percentage of all biopsies
Liver	10	9.5	4.4
Kidney	1	5.3	0.4
Pancreas	5	17.2	2.2
Spleen	1	25.0	0.4
Adrenal gland	1	14.3	0.4
Retroperitoneum	4	9.3	1.8
Other	1	5.6	0.4

Among the 88 lesions that were suspected of being primary lesions, the histological diagnosis showed a primary malignant tumor in 48 (54.5%), a benign lesion in 34 (38.6%), and a metastatic lesion in 6 (6.8%). Among the 137 lesions that were suspected of being metastatic lesions, the histological diagnosis confirmed that suspicion in 84 (61.3%), whereas it showed a benign lesion in 39 (28.5%) and a primary malignant tumor in 14 (10.2%).

## DISCUSSION

The results of the present study demonstrate that CT-guided percutaneous biopsy of abdominal lesions is an effective procedure with a low rate of complications, regardless of the indications, lesion characteristics, and technique employed. Our study showed that this type of biopsy has an accuracy of nearly 90%, taking into consideration the abdominal cavity as a single group and a complication rate of less than 10%. Among the 225 procedures analyzed, there was only one procedure-related death (i.e., a procedure-related mortality rate of 0.4%). The histological result was discordant with the diagnostic hypothesis in approximately 45% of the cases in which a primary lesion was suspected and in 39% of those in which a metastatic lesion was suspected, demonstrating that this procedure can have a major impact on the management of cancer patients. In all of the patients who underwent a second percutaneous procedure due to an insufficient sample in the first biopsy, the diagnostic hypothesis was confirmed.

The choice of the imaging modality is based on a number of factors^(^^13^^)^: physician experience; the size and location of the lesion; the possible access routes; the visualization capacity; and the availability and cost of the equipment. Ultrasound guidance can be used in the biopsy of many abdominal lesions, including masses in the liver, pancreas, or kidney, as well as bulky lymphadenopathy and large adrenal masses. Its advantages include real-time imaging, multiplanar evaluation capability, which facilitates complex angular approaches often required for biopsies in the upper abdominal quadrant, and the absence of ionizing radiation. Disadvantages include impaired visualization of deep lesions and obscuration caused by interposition of intestinal gases or bones. CT-guided biopsy is used for abdominal lesions that are not well characterized by ultrasound. The advantages of the CT-guided procedure are the high resolution of the image, the ability to visualize the intestine, and the better characterization of retroperitoneal structures. Disadvantages include additional radiation exposure for the patient, a lack of real-time feedback during needle advancement/biopsy, and difficulty in using the angular approaches required for masses in the upper abdomen, near the diaphragm^(^^13^^-^^15^^)^.

The most common type of biopsy in our study was liver biopsy, which is considered a safe and effective procedure for the diagnosis of liver diseases. In keeping with the findings of other studies in the literature, we have shown that imaging-guided percutaneous biopsies, performed by the coaxial technique, are safe and present a minimal risk of severe complications. The complication rates observed in the literature vary from less than 1% to 4%. Among the 105 liver biopsies evaluated in the present study, complications occurred after six (5.7%), the complication necessitating hospitalization in only one of those six cases. These differences in complication rates may be related to some factors. In most studies, patients who undergo liver biopsies are not routinely submitted to follow-up tomography after the procedure, remaining under clinical observation only^(^^16^^-^^18^^)^. At our facility, follow-up tomography scans are acquired 1-2 h after every liver biopsy, to rule out complications, and the patients remain under clinical observation in the period between imaging studies. In the present study, our analysis also included minor complications without clinical repercussions, such as self-limited bleeding in the subcapsular region and abdomen, which are not counted as complications in most studies. Another factor that we should consider is the fact that our liver biopsies are usually performed by residents in our interventional radiology fellowship program, under supervision.

The second most common type of biopsy in our study was retroperitoneal biopsy, which is consistent with the findings of other studies in the literature. Lesions with difficult-to-access retroperitoneal locations can be biopsied using supplementary techniques, and our study did not show a significant difference between the patients in whom such techniques were used and those in whom direct access was used, in terms of the rate of complications. Among the retroperitoneal lesions evaluated in the present study, the most common was retroperitoneal lymphadenopathy, which was seen in 43 cases. The safety of CT-guided percutaneous biopsy of these lesions is well documented. We found that the complication rate among such biopsies was 2.3%, even lower than the 9.4% reported by Tomozawa et al.^(^^7^^)^, indicating how safe this type of procedure is^(^^9^^,^^11^^)^. The second most common retroperitoneal lesion in our sample was pancreatic lesion, seen in 29 cases. Most studies of CT-guided percutaneous biopsy have shown that the procedure has a diagnostic accuracy of over 90% for pancreatic lesions. We found a slightly lower rate: 82.8% of the results obtained in our study were sufficient for the diagnosis. In the present study, the complication rate for CT-guided percutaneous biopsy was approximately 10%, compared with the 0-5% reported in the literature, including an earlier study conducted at our center. That difference is attributable to the fact that most studies consider only the major complications for this type of procedure^(^^19^^,^^20^^)^.

In the present study, 19 renal biopsies were performed, and the biopsy sample was insufficient for the diagnosis in only one (5.2%), which translates to an accuracy rate of 94.8%. Among other studies in the literature, the diagnostic success rate varies from 77% to 92%^(^^21^^,^^22^^)^.

This study has some limitations. Because it was a retrospective study, we were able to evaluate only those patients for whom the necessary information was available in the hospital data system. In addition, some of the images obtained during the procedures were lost during the transition to a new data management system. Although CT-guided percutaneous biopsies of abdominal lesions are already part of the routine at our center, this survey helped corroborate what we have observed in clinical practice. Future studies evaluating specific procedures or indications may help further improve our results.

## CONCLUSION

The present study demonstrated that the most common indication for CT-guided percutaneous biopsy was the suspicion of a metastatic lesion. In more than one third of those cases, the result of the biopsy was different from the clinical suspicion. All procedures were performed with the coaxial technique, and the use of additional techniques for difficult-to-access abdominal masses did not increase the complication rate. The results demonstrated a diagnostic accuracy of approximately 90%, with a low rate of complications, the most common complication being self-limiting bleeding. We identified no clinical, radiological, or procedure-related factors that had a statistically significant impact on the success or complication rates of CT-guided percutaneous biopsies of abdominal lesions.
